# HPLC/UV approach method for the first simultaneous estimation of molnupiravir and ertapenem as a binary mixture in human plasma and dosage form as a regimen for COVID-19 treatments

**DOI:** 10.1186/s13065-023-01024-y

**Published:** 2023-09-21

**Authors:** Khaled K. Afify, Ramadan Ali, Mohammad A. El-Dosoky, Mohamed wafaa I. Nassar

**Affiliations:** 1 Pharmaceutical Analytical Chemistry Department, Faculty of Pharmacy, Al-Azhar University, Assuit branch, 71524 Assuit, Egypt; 2 Department of Pharmaceutical Chemistry, Faculty of Pharmacy, University of Tabuk, 71491 Tabuk, Saudi Arabia; 3 Pharmaceutical Analytical Chemistry Department, Faculty of Pharmacy, Al-Azhar University, 11751 Nasr City, Cairo Egypt

**Keywords:** HPLC-UV, Human plasma, MOL, ERT, COVID-19

## Abstract

COVID-19 is a serious virus that can have a lot of effects, one of which is a secondary bacterial infection that can be more life-threatening and even lethal than the initial viral infection. Hence a fast and sensitive HPLC/UV method was developed and validated for the first estimation of a binary mixture of molnupiravir (MOL) and ertapenem (ERT) as a co-administrated medicine for the management of COVID-19 in pharmaceutical dosage forms, and human plasma samples. The drug combination was separated within 5 min via RP-ODS column using isocratic elution with a mobile phase of 0.05 M phosphate buffer (pH 3.5): acetonitrile with a 76: 24% ratio v/v. The presented method provided a linear response ranging from 0.03 to 17.0 and 0.05–20 µg mL^−1^ with LOD values of 0.009 and 0.008 µg mL^−1^ for MOL and ERT respectively. The good separation and high sensitivity of the HPLC method provide the determination of the cited drugs in human plasma without matrix interference with a percent of recovery ranging from 94.97 ± 2.05 to 98.44 ± 1.92. Based on the results, this method could be utilized to monitor cited drugs in quality control and therapeutic laboratories.

**Supplementary Information:**

The online version contains supplementary material available at 10.1186/s13065-023-01024-y.

## Introduction

**Fig. 1:**
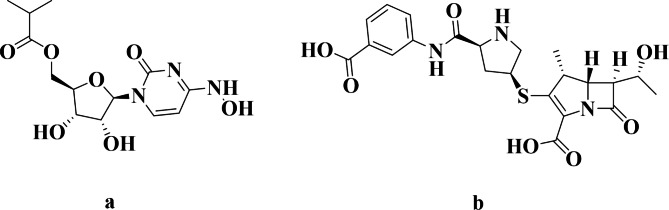
Chemical structure of studied drugs **(a)** MOL and **(b)** ERT.

At the end of 2019, numerous cases of patients suffering from mild to severe symptoms that are similar to bacterial pneumonia without any known cause were reported in Wuhan, Hubei province, China [1]. An outbreak of the infection resulted in thousands and millions of cases worldwide. The unidentified pneumonia was defined to be caused by a novel coronavirus (CoV) named 2019-nCoV [2, 3]. On February 11, 2020, the International Committee of Taxonomy of Viruses (ICTV) named this novel coronavirus as SARS-CoV-2 [4]. In March 2020, the World Health Organization (WHO) declared the COVID-19 virus a global pandemic [5]. Plenty of efforts around the world have been directed toward developing therapeutic strategies and instructions to decrease the spread of infection and disease symptoms [6]. Although the reported total COVID-19 deaths for the two most recent years were around 5.94 million worldwide, an estimated 18.2 million people died from COVID-19 disease during this period. This could be due to poor reporting, insufficient testing facilities, reduced access to healthcare services, and even political considerations [7]. For control of the health hazards of COVID-19 disease, an antiviral drug directly acting on coronavirus was urgently needed. MOL (Fig. 1**)** chemically is N-Hydroxy-5’-O-isobutyryl-3,4-dihydrocytidine [(2R,3 S,4R,5R)-3,4-Dihydroxy-5-[4-(hydroxyamino)-2-oxopyrimidin-1-yl] oxolan-2-yl] methyl 2-methylpropanoate. MOL is a broad-spectrum antiviral prodrug that is rapidly metabolized in vivo into the active triphosphate form, inhibiting viral RNA polymerase, which is necessary for viral replication [8]. MOL is the first oral, direct-acting antiviral that is highly effective at reducing nasopharyngeal SARS-CoV-2 infectious virus and viral RNA with a favorable tolerability and safety profile [9]. The severity of COVID-19 disease is not only attributed to the viral invasion but also to the secondary bacterial infection that can be more life-threatening and even lethal than the initial viral infection [10]. So, the need for a wide-spectrum antibiotic oriented to face the bacterial infection that may have emerged after or just before the COVID-19 infection is essential.

Ertapenem sodium (Fig. 1) is the monosodium salt of (4R,5 S,6 S)-3-[(3 S,5 S)-5-[(3-carboxyphenyl) carbamoyl] pyrrolidin-3-yl] sulfanyl-6-(1-hydroxyethyl)-4- methyl-7-oxo-1- azabicyclo [3.2.0] hept-2-ene-2-carboxylic acid. It is a β-lactam antibiotic of the carbapenem class with an exceptionally broad spectrum of activity [11]. ERT has the advantage of being a broader spectrum of activity than other beta-lactams like penicillins and cephalosporins and is more resistant to the enzyme β-lactamase which is the main cause of resistance of many bacteria [12]. In the covid-19 pandemic period, daily inpatient ERT therapy can be an alternative to hospitalization for the treatment of complicated urinary tract infections, which is safe and cost-effective [13]. The need for an effective antibiotic in covid-19 patients for treating a complication of the covid-19 disease concomitant with an antiviral is so essential. ERT combined with cefazolin resulted in successful sterilization of blood cultures within 24 h of administration in a covid-19 patient previously bacteremic for more than 10 days [14]. Co-administration of MOL as an antiviral drug with ERT as an antibacterial drug for secondary bacterial infection for a covid-19 patient is crucial. Till the time of writing this manuscript, there is no reported method for the determination of both MOL and ERT as possible co-administered drugs in some cases related to covid-19 patients.

The literature review revealed only a few methods for determining the cited drugs alone or in combination with other drugs, such as HPLC methods [15–26], HPTLC methods [27], electro-analytical methods [28–30], spectrophotometric [31–37], and spectrofluorimetric [3, 38–40].

The presented method describes a simple, rapid, sensitive, reliable, and cost-effective HPLC method for simultaneous estimation of MOL and ERT in their pharmaceutical formulations and human plasma samples which could be helpful in clinical and therapeutic laboratories.

## Experimental

### Instrumentation and software

The HPLC separation of the MOL and ERT mixture was carried out using Waters 717 Instrument, the instrument connected with an autosampler with a sample thermostat that contains Alltech, 426 LC pump, and UV/VIS detector (Waters Millipore, USA). The results were obtained using Kromex (Estonia) software.

### Chemicals and standard solutions

MOL (analytical standard, purity 99.96%) was mercifully given by the Egyptian International Pharmaceutical Industries Co. (EIPICo., Egypt). Molcovir® capsules (containing 200 mg MOL per capsule; batch number: MOLCD1003D) were obtained from Optimus Pharma, India.

ERT (analytical standard, purity 99.89%) and Invanz® vial (containing 1000 mg ERT per vial; batch number: W011981) were obtained from Merck Sharp & Dohme, USA. All the solvents used were of HPLC grade. Acetonitrile (ACN), sodium di-hydrogen phosphate, and 85% orthophosphoric acid were HPLC grade obtained from EL-Nasr Co., Egypt.

### Standard drug solutions preparation

The standard stock solutions of concentration 1.0 mg mL^− 1^ for each drug were made by transferring accurately weighed 25 mg of authentic powder into a 25-mL calibrated flask then diluted with about 15 ml double distilled water and sonicated for about 5 min. The flask was completed to 25 ml by double distilled water to obtain a stock solution of a concentration of 1.0 mg mL^− 1^, then stored in the refrigerator at 4ºC. The working solutions of MOL and ERT were prepared by diluting the standard stock solution (1.0 mg mL^− 1^) by the mobile phase using volumetric flasks to obtain working solutions within the concentration range of 0.03–20 µg mL^− 1^.

### The chromatographic conditions

Twenty microliters of working solution for the specified drugs in the concentration range of 0.03–20 µg mL^− 1^, were injected into the HPLC-UV system. The separation and quantitation were carried out using a GL Science RP-ODS column (25 cm x 4.6 mm id, 5 μm particle size) (Japan) as a stationary phase. The mobile phase consists of 0.05 M phosphate buffer (pH 3.5 adjusted by 85% ortho-phosphoric acid) and acetonitrile in the ratio of 76: 24% (v/v). The flow rate of the mobile phase in this method was set to 1 mL min^− 1^ and the eluted drugs were detected using a UV detector at 230 nm. The separation and quantitation were performed in this method at ambient temperature.

### Pharmaceutical dosage form preparation

In order to analyze the studied drugs, ten capsules of Molcovir® were evacuated and mixed well then, an equivalent amount of 25 mg of the studied drug was transferred into a 25 mL volumetric flask containing ultra-pure water. The solution was filtrated through a 0.45-µm cellulose acetate membrane and diluted using the mobile phase for the preparation of working solutions in the linearity range of the calibration.

Invanz® vial was evacuated and mixed well and then an equivalent amount of 25 mg of the studied drug was put into a 25 mL volumetric flask containing ultra-pure water, filtered, and diluted in the same way previously mentioned to obtain working solutions with concentrations in the linearity range of the calibration.

The mixture of working solutions for the binary mixture was prepared using the mobile phase.

### Analysis of the studied drugs in human plasma

Human plasma samples were collected from five healthy volunteers aged 22–35 years into heparinized tubes, in accordance with the responsible committee on human experimentation’s (institutional and national) ethical guidelines and the Helsinki Declaration of 1975, as revised in 2008. The method and the study were approved by the Egyptian Network of Research Ethics Committees (ENREC). After collecting plasma samples, 1.0 mL of plasma samples were spiked with different concentrations of the working standard solution (5, 50, 100, 150, and 170 µg mL^− 1^). For protein precipitation, 1.0 mL of acetonitrile was added [3]. After vortex mixing, the supernatant solution was separated and diluted in 10 ml volumetric flasks with the mobile phase to obtain final concentrations (0.5, 5.0, 10, 15.0, and 17.0 µg mL^− 1^). The final solution was centrifuged afterward for 20 min at 4000 rpm. A 0.45 μm cellulose acetate membrane was used for filtration of the supernatant solution, then 20 µL of supernatant was injected into the HPLC-UV system.

## Results and discussion

**Fig. 2:**
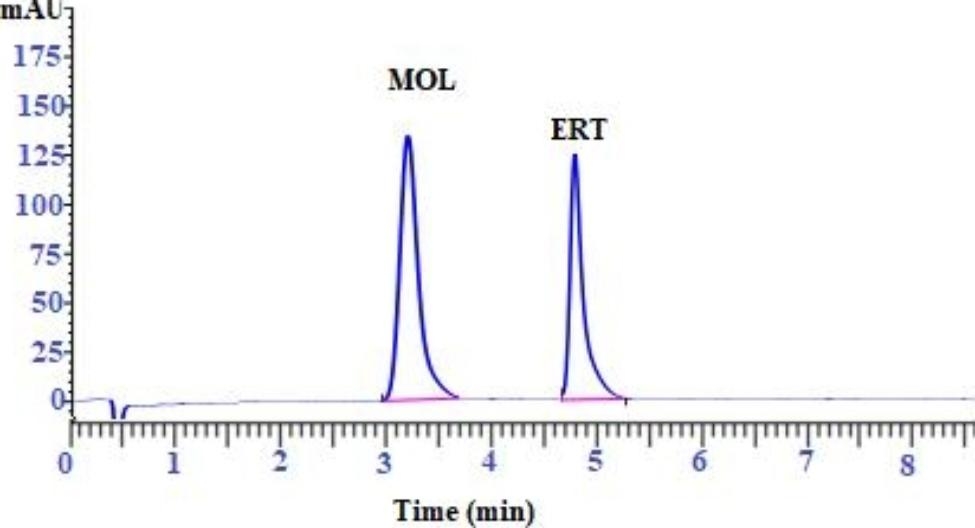
HPLC Chromatogram for separation of studied drugs (5 µg mL^− 1^ for each)

The goal of this study is to create a rapid, simple, and sensitive HPLC method for the simultaneous quantification of MOL and ERT as a binary mixture in pure form and human plasma for the first time (Figure 2).

Various system suitability factors including capacity factor (**k’)**, retention time (**t**_**R**_), resolution **(R**_**s**_**)**, separation factor (**α)**, tailing factor (T), and number of theoretical plates **(N)** were investigated to assess the system performance and the method repeatability for separation of cited drugs. MOL and ERT were separated simultaneously after 3.2 and 4.86 min, respectively (Figure 2).

The summarized results in Table 1 revealed a good separation between MOL and ERT, where MOL and ERT were separated at 3.2 and 4.86 min respectively having tailing factor values of 1.09 and 1.05 for MOL and ERT respectively. The capacity factor (**K’)** was found to be 7 and 11.15 for MOL and ERT respectively. The column efficiency was studied by calculating the resolution (**R**_**S**_). It was found that R_s_ between MOL and ERT was equal to 2.3 with a selectivity factor equal to 1.53 which refers to a good separation of the two studied drugs (Table 1).

**Table 1:** System suitability parameters for the studied drugs using HPLC-UV method

Parameters	MOL	ERT
Retention time (min), t_R_	3.2 ± 0.1^a^	4.86 ± 0.13^a^
Void time (min)	0.4	0.4
Adjusted Retention time (min), t_R_^`^	2.8	4.46
Capacity factor, K’	7	11.15
Number of theoretical plates (N, plates)	1820 ± 3.5	3893.76 ± 20.21
Height equivalent theoretical plate (HETP, cm/plate)	0.013	0.006
Tailing factor (T)	1.09	1.05
Resolution (Rs)		2.3
Selectivity factor, α		1.53

(^a^): SD

### Optimization of HPLC variables

For achieving the most suitable drug separation, different factors which influence separation time and peak symmetry were investigated to choose the most appropriate conditions. These factors include mobile phase composition, flow rate, and buffer concentration. Each factor was assessed individually while others remain unchanged.

#### Mobile phase composition

To obtain the most optimum drugs separation in a suitable time giving reliable peak area, various mobile phases composition was tried as acetonitrile (ACN): methanol, water: methanol, and ACN: water with varying percentages, and it was observed that no good separation of studied peaks was achieved with these trials. Therefore, the mobile phase that consists of a mixture of 0.05 M phosphate buffer (pH 3.5 adjusted by 85% ortho-phosphoric acid), acetonitrile (76: 24% v/v), was found to be the optimum mobile phase for separation as in Fig. 2. As it is observed from Fig. 1, MOL contains four hydrogen bond donors and seven hydrogen bond acceptors, enabling it to form four hydrogen bonds at least with orthophosphoric acid. Also, ERT contains five hydrogen bond donors and nine hydrogen bond acceptors, which easily bind with orthophosphoric acid. This makes the forcing power for both drugs elution.

ERT has a higher retention time than MOL due to the presence of lipophilic phenyl moiety in ERT which decreases polarity in comparison to MOL as well as log p of ERT (-1.8) is more than the log p of MOL (−0.8) [41, 42].

Besides, the effect of the flow rate was further studied to achieve sharp symmetric peaks of the cited drugs within a reasonable time. It was found that flow rates from 0.9 to 1.1 ml/min showed good separation between the MOL and ERT with sharp symmetric peaks, so 1 mL/min was the suitable flow rate.

#### Buffer PH optimization

**Fig. 3:**
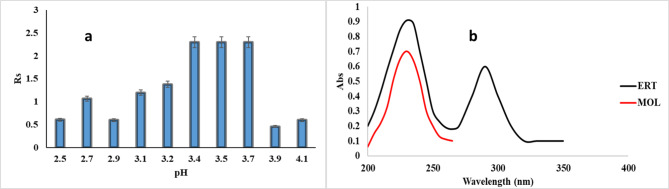
**(a)** effect of pH range for separation MOL and ERT mixture (5 µg mL^− 1^ for each), **(b)** UV spectrum for studied drugs

The buffer PH of 0.05 M phosphate buffer was checked in the range from 2.5 to 4.1 to obtain good separation for MOL and ERT (Fig. 3a), after comparing it was observed that pH 3.5 was selected as the optimum pH for the separation of the studied drugs.

PH 3.5 was chosen according to the pKa of both drugs which is 2.2, 10.2, and 12 for MOL, 3.2 and 9 for ERT, so the use of buffer to maintain the best ionization and solubility of both drugs above pKa 2 and below 10.

Also, the wavelength for the cited drugs was estimated using a Shimadzu spectrophotometer, and the UV spectra of the cited drugs were recorded at 230 nm (Fig. 3b).

### Validation of the presented HPLC method

The HPLC method was validated based on International Conference on Harmonization (ICH) guidelines [39].

#### Linearity and calibration curve

To obtain the calibration curve for each drug, the peak area for each concentration in the range of (0.03–17.0 µg mL^− 1^) for MOL and (0.05–20.0 µg mL^− 1^) for ERT was plotted against the corresponding peak area, and the range specified for each drug was observed to produce a linear relationship with high correlation coefficient.

The linearity range and the sensitivity parameters were summarized in Table 2.

**Table 2:** Quantification parameters for HPLC/UV method for determination of studied drugs

Parameters	MOL	ERT
Wavelength (nm)	230	230
Flow rate (mL/min)	1.0	1.0
Linearity range (µg mL^− 1^)	0.03–17.0	0.05–20.0
Correlation coefficient (r)	0.9994	0.9991
Determination coefficient (r^2^)	0.9995	0.9993
Intercept ± SD	78,834 ± 40.56	61,541 ± 30.22
Slope ± SD	14 × 10^3^ ± 2291.12	12 × 10^3^ ± 901.10
LOD (µg mL^− 1^)	0.009	0.008
LOQ (µg mL^− 1^)	0.02	0.02

#### Accuracy

The accuracy of the proposed approach was evaluated using five distinct concentration levels (0.5,5.0, 10.0, 15, and 17.0 µg mL-1) by injecting each concentration three times on the HPLC-UV system, for the drugs under investigation. The results were summarized in Table 3 which refers to the high accuracy of the proposed method.

**Table 3:** Evaluation of the accuracy and precision of the HPLC method for estimation of the cited drugs

		MOL				ERT			
		Intra-day assay		Inter-day assay		Intra-day assay		Inter-day assay	
Sample no.	Taken (µg mL^− 1^)	Found (µg mL^− 1^)	% Recovery* ± RSD	Found (µg mL^− 1^)	% Recovery*± RSD	Found (µg mL^− 1^)	% Recovery* ± RSD	Found (µg mL^− 1^)	% Recovery*± RSD
1	0.5	0.51	101.65 ± 0.19	0.52	102.60 ± 1.19	0.49	99.16 ± 1.01	0.48	96.54 ± 1.81
2	5	4.99	99.83 ± 0.70	5.09	101.87 ± 2.16	5.01	100.39 ± 0.94	5.12	102.44 ± 2.15
3	10	9.98	99.80 ± 0.21	10.13	101.33 ± 1.40	10.14	101.43 ± 0.54	10.15	101.50 ± 1.77
4	15	15.40	102.66 ± 0.34	15.11	100.71 ± 1.74	15.06	100.40 ± 0.63	14.97	99.78 ± 0.85
5	17	17.20	101.17 ± 0.90	17.28	101.67 ± 1.02	17.05	100.29 ± 0.44	17.17	100.59 ± 0.88

*: Average of three determinations

#### Repeatability and intermediate precision

The intra-day repeatability was evaluated as RSD using 5 different concentrations for each drug, each analyzed three replicates (n = 3) on the same day, and the intermediate precision was checked by analyzing the same 5 different concentrations on three consecutive days. RSD was found to range from 0.19 to 2.16 which shows acceptable repeatability and intermediate precision.

#### The limit of detection (LOD), the limit of quantitation (LOQ), and the sensitivity

According to ICH guidelines recommendations [43], the lower limit of detection (LOD) and lower limit of quantitation (LOQ) were calculated using the following equations:

1 $$$$LOD = 3.3\sigma /S$$$$

2 $$$$LOQ = 10\sigma /S$$$$

Where ϭ is the standard deviation of the response and S is the slope.

The calculated results were summarized in Table 2, which refers to the high sensitivity of the HPLC method compared to other reported methods [15–24]. For the reason of high sensitivity, the proposed HPLC method gives great value in the determination of the studied drugs in plasma samples.

#### Robustness

To examine the robustness of the proposed HPLC method, one experimental variable was varied independently while the others were kept constant. The variables examined were mobile phase composition ratio, buffer concentration, value of pH, mobile phase flow rate, and detection wavelength. To check the effect of mobile phase ratio change, 74/26 v/v, and 78/22 v/v ratios were checked. The pH of the buffer solution was checked at 3.4 and 3.6 pH values and the buffer concentration was changed to 0.04 and 0.06 M. As shown in Table 4 these slight modifications of the separation system parameters have no significant effect on the results of the method revealing method robustness.

**Table 4:** Robustness of the proposed method for estimation of the studied drugs using (10 µg mL^− 1^)

Parameters	MOL	ERT
	% Recovery ± RSD^*^	% Recovery ± RSD^*^
No variations	100.10 ± 0.65	100.50 ± 0.90
Mobile phase		
74/26 v/v 78/22 v/v	98.50 ± 0.79 99.65 ± 0.66	98.80 ± 1.34 98.93 ± 0.61
Wavelength (nm)		
225 235	99.75 ± 0.82 99.54 ± 0.22	99.86 ± 1.22 99.98 ± 0.77
Flow rate (mL/min)		
0.9 1.1	99.22 ± 0.54 98.76 ± 0.33	98.41 ± 0.44 98.27 ± 0.63
pH		
3.4 3.6	99.88 ± 0.43 99.93 ± 0.79	99.33 ± 0.70 99.25 ± 0.91
Buffer concentration (M)		
0.04 0.06	99.15 ± 1.32 99.23 ± 1.05	99.26 ± 1.45 98.12 ± 0.33

*Average of three determinations

#### Linearity, accuracy, and precision in human plasma

The proposed method was applied for the determination of the studied drugs in human plasma samples, and it was observed to give a linear relationship between spiked drug concentration and the corresponding peak area in the range of (0.03–17.0 µg mL^− 1^) for MOL and (0.05–20.0 µg mL^− 1^) for ERT. The value of both LOD and LOQ were calculated using the equations previously mentioned in Sect. 3.2.4. The LOQ for MOL and ERT was found to be 0.04 and 0.05 µg mL^− 1^ respectively and the LOD was found to be 0.013 and 0.015 µg mL^− 1^ for MOL and ERT respectively.

According to US-FDA criteria [44], the explored approach underwent bio-analytical validation, where the accuracy and precision were examined in human plasma. Using low-quality control sample (LQC), medium-quality control sample (MQC), and high-quality control samples (HQC) for MOL and ERT, the three concentration points were analyzed at the same day (intra-day) where n = 6, and inter-daily (n = 9). According to the results listed in Table S1, the application of the proposed method for analysis of the studied drugs in human plasma exhibits good repeatability precision, as the percent RSD ranges from 1.32 to 2.50 and from 1.44 to 2.20 for MOL and ERT respectively, and the percent recovery ranging from 95.01 to 97.10%.

#### Matrix effect and selectivity

**Fig. 4:**
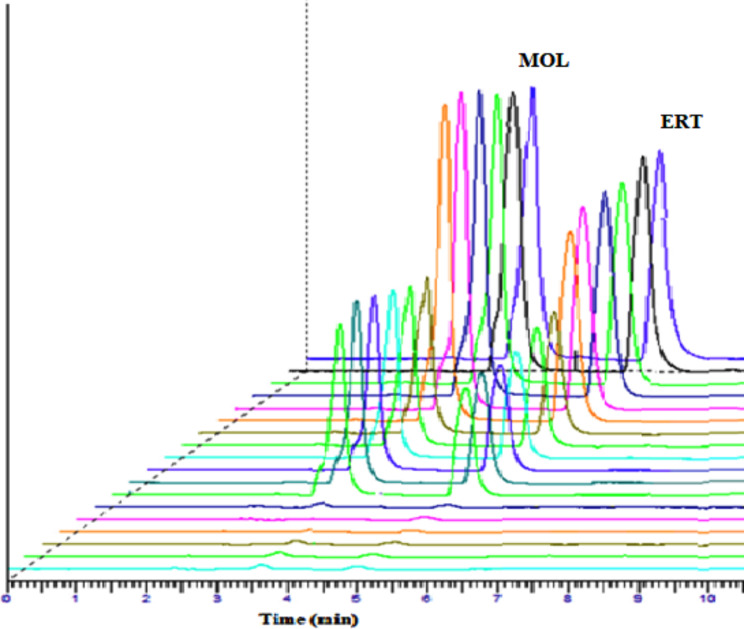
3D Chromatogram for matrix effect using the synthetic mixture in human plasma (0.5, 5.0, and 15.0 µg mL^− 1^ for each)

For evaluation of the method selectivity, three points of quality control samples (low-quality control sample (LQC), medium quality sample (MQC), and high-quality control sample (HQC) were examined. Those concentrations were (0.5, 5.0, and 15.0 µg mL^− 1^) for both MOL and ERT that were used to examine the possible plasma matrix effect in human plasma samples on the determination of medications under investigation. The recovery was discovered to range from 95.55 ± 1.86 to 97.40 ± 2.44 as in Fig. 4. The results refer to the absence of a significant plasma matrix with the tested regimen as a binary mixture for treatment of COVID-19, which validates the excellent selectivity of the suggested technique as depicted in Fig. 4.

#### Stability

The stability of the studied analytes was assessed under different experimental conditions, resembling plasma sample storage and preparation until HPLC analysis. QC samples at three concentration levels (LQC, MQC, and HQC) were assessed under the following conditions: (1) Short-term stability for 12 h at -20 °C, (2) Long-term stability for 15 days at -20 °C, (3) post-preparative stability for 6 h at room temperature 25 °C, (4) Three Freeze–thaw cycle stability at -20 °C. The results obtained from the tested samples at these different conditions revealed that the percentage of recovery was between 85 and 115% which indicates the accepted stability of the studied drugs under the studied different conditions (Table 5).

**Table 5:** Stability of the cited drugs in human plasma samples under different conditions

	LQC 0.1 µg mL^− 1^	MQC 5 µg mL^− 1^	HQC 15 µg mL^− 1^
**MOL**			
Short-term stability for 12 h (-20 °C)	98.57 ± 1.85	98.12 ± 1.87	98.75 ± 2.01
Long-term stability for 15 days at (-20 °C)	97.10 ± 2.20	96.54 ± 2.34	97.91 ± 1.65
Post-preparative stability (6 h at room temperature 25 °C)	96.33 ± 1.52	98.25 ± 2.84	97.48 ± 1.79
Three Freeze-thaw cycle stability (-20 °C)	98.65 ± 1.78	97.62 ± 1.92	97.50 ± 2.19
**ERT**			
Short-term stability for 12 h (-20 °C)	98.30 ± 1.53	97.85 ± 1.73	97.65 ± 2.15
Long-term stability for 15 days at (-20 °C)	96.52 ± 1.72	97.85 ± 1.62	97.05 ± 2.50
Post-preparative stability (6 h at room temperature 25 °C)	97.82 ± 2.01	96.74 ± 1.59	98.12 ± 1.47
Three Freeze-thaw cycle stability (-20 °C)	96.94 ± 1.95	97.15 ± 2.37	96.38 ± 2.45

Data presented as recovery (%) ± SD (n = 5)

### Applications of the chromatographic method

#### Estimation of MOL and ERT in their pharmaceutical forms

The developed HPLC method was utilized for the determination of the studied drugs in their dosage forms (Molcovir capsules® and Invanz vial®). The percentage of recovery obtained by the proposed method was determined to be 101.50 ± 0.92 and 101.33 ± 0.53 respectively and was compared with that of the reported method [3, 23]. All the results summarized in Table 6 refer to the high accuracy of the proposed HPLC method.

**Table 6:** Pharmaceutical determination of studied drugs compared with reported methods

Dosage form	%Recovery ± SD ^a^		*t-*value ^b^	*F-*value ^b^
	Proposed	Reported ^b^		
Molcovir capsules® [3]	101.50 ± 0.92	100.11 ± 1.42	1.50	3.11
Invanz vial® [23]	101.33 ± 0.53	100.72 ± 1.11	1.42	2.31

^a^ mean of five determinations, ^**b**^the tabulated *t-* and *F-* values at 95% confidence limit are 2.78 and 6.39, respectively

#### Applications of HPLC in spiked human plasma

The new method’s high sensitivity enables the detection of MOL and ERT medicines in spiked human plasma without matrix interference as a synthetic mixture (Fig. S1). For the tested procedures at five different concentration levels applied, the recovery percentage was discovered to be between 94.97 and 98.44%, as indicated in Table 7. For the examined medicines, the percent RSD values fall between 1.55 and 2.70 respectively. The data obtained revealed that the studied drugs can be determined in plasma samples without the interference of the matrix effect.

**Table 7:** Analysis of studied drugs in spiked human plasma using the proposed HPLC-UV method

	MOL	ERT
Added conc. (µg mL^− 1^)	% Recovery^*^ ± RSD	% Recovery^*^ ± RSD
0.5	97.39 ± 2.14	95.19 ± 2.14
5.0	97.67 ± 1.55	97.47 ± 1.66
10.0	98.37 ± 1.73	98.21 ± 1.73
15.0	98.44 ± 1.92	97.54 ± 1.92
17.0	94.97 ± 2.05	95.29 ± 2.70

*: Average of three replicates

#### Comparison study between the presented method and reported methods

Comparing the results in our work with other reported as in Table 8. It was found the presented work can serve as a probe for the first simultaneous estimation of a binary mixture of MOL and ERT with low concentration with higher sensitivity and reliability than other reported methods.

**Table 8:** Comparison between reported methods and the proposed method for MOL & ERT.

Method	MOL		Ref.	ERT		Ref.
	LOD µg mL^− 1^	LOQ µg mL^− 1^		LOD µg mL^− 1^	LOQ µg mL^− 1^	
HPLC	0.009	0.02	Proposed method	0.008	0.02	Proposed method
HPLC	0.06	0.21	[15]	2.8	8.44	[18]
TLC	1.21	3.66	[27]	11.74	35.60	[20]
Spectrophotometry	1.0	1.5	[36]	2.0	6.06	[18]

## Conclusion

This study aims to the first simultaneous estimation of a binary mixture of MOL and ERT as a possible treatment regimen in COVID-19 infection. It has been successively applied to the analysis of the studied drugs in their commercial dosage forms and human plasma samples. The proposed method appears to be sensitive (LOD is 0.009 µg mL^− 1^ and 0.008 µg mL^− 1^ for MOL and ERT respectively), selective, rapid, and relatively low cost which facilitates its application in quality control units. Also, the high sensitivity and selectivity obtained after application of the proposed method on analysis of the studied drugs in human plasma samples gives the advantage of being applied for drug analysis in clinical units.

**Declaration section**.

### Electronic supplementary material

Below is the link to the electronic supplementary material.

Supplementary Material 1

## Data Availability

All data generated or analyzed during this study are included in this published article [and its additional files].
