# Supplementary material for: HPLC/UV approach method for the first simultaneous estimation of molnupiravir and ertapenem as a binary mixture in human plasma and dosage form as a regimen for COVID-19 treatments
Source: BMC Chem. 2023 Sep 21;17(1):121. doi: 10.1186/s13065-023-01024-y (PMC10515236; doi:10.1186/s13065-023-01024-y)
Supplement: Supplementary file 1 — Supplementary Material 1 [file 13065_2023_1024_MOESM1_ESM.docx]

**HPLC/UV approach method for the first simultaneous estimation of molnupiravir and ertapenem as a binary mixture in human plasma and dosage form as a regimen for COVID-19 treatment**

Khaled K. Afify^*a^, Ramadan Ali ^a^, Mohammad A. El-Dosoky ^b^, Mohamed wafaa I. Nassar ^b^

^a^ Pharmaceutical Analytical Chemistry Department, Faculty of Pharmacy, Al-Azhar University, Assuit branch, assuit 71524, Egypt.

^b^ Pharmaceutical Analytical Chemistry Department, Faculty of Pharmacy, Al-Azhar University, 11751, Nasr City, Cairo, Egypt.

***Corresponding Author:** [khaledafify@azhar.edu.eg](mailto:khaledafify@azhar.edu.eg)

**Table S1** Accuracy and precision of the proposed method for determination of studied drugs in human plasma.

| **Inter-day assay (n=9)** | | **Intra-day assay (n=6)** | |  | |
| --- | --- | --- | --- | --- | --- |
| **Precision**  **(%RSD)** | **Accuracy**  **(%Recovery)** | **Precision**  **(%RSD)** | **Accuracy**  **(%Recovery)** | **Taken**  **(µg mL^-1^)** |  |
| 1.70 | 95.33 | 1.40 | 97.10 | **MOL**  **0.1** |  |
| 1.65 | 96.01 | 2.50 | 96.87 | **5** |  |
| 1.32 | 97.03 | 1.72 | 97.00 | **15** |  |
|  |  |  |  | **ERT** |  |
| 2.06 | 95.01 | 1.44 | 96.09 | **0.1** |  |
| 2.05 | 96.45 | 1.84 | 96.00 | **5** |  |
| 2.20 | 96.33 | 2.03 | 96.08 | **15** |  |


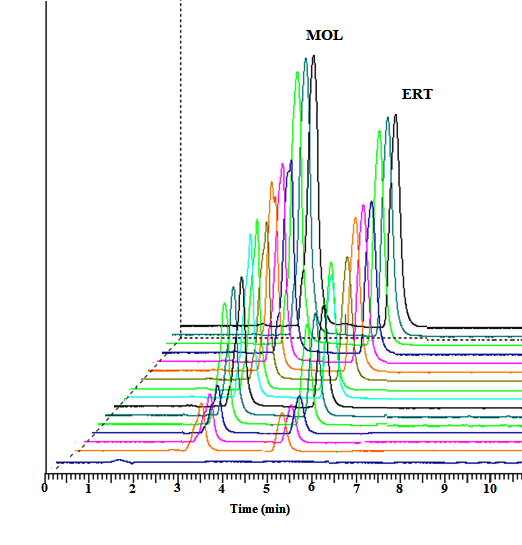


**Fig. S1** 3D chromatogram for a mixture of ertapenem and molnupiravir in human plasma
